# Evaluating distal renal tubular acidification function in primary hyperparathyroidism and its effects on bone mineral density

**DOI:** 10.1186/s12891-021-03954-x

**Published:** 2021-01-12

**Authors:** Wangna Tang, Hongwei Jia, Baoping Wang, Yun Chai, Tong Liu, Hao Wang, Chenlin Dai

**Affiliations:** Endocrinology and Metabolism Disease Department, Tianjin Medical University General Hospital, 154# Anshan Road, Heping District, 300052 Tianjin, China

**Keywords:** Primary hyperparathyroidism, Distal renal tubule, Acidification function, Bone mineral density

## Abstract

**Background:**

Primary hyperparathyroidism (PHPT) is a common endocrinopathy that may increase fracture risk and decrease bone mineral density (BMD). Some patients develop distal renal tubular acidification dysfunction under conditions of hyperchloraemia or hyperchloraemic acidosis. To examine whether this dysfunction influences the clinical outcome, we explored the distal renal tubular acidification function in patients with PHPT and its effects on the clinical manifestations of the disease.

**Methods:**

We retrospectively analysed 75 PHPT patients with regard to renal tubular acidification and blood gas analysis. The patients were divided into two groups, the renal tubular acidification dysfunction group and normal function group.

**Results:**

Serum phosphate level and total hip bone density were significantly decreased and 25OHD level was significantly increased in the renal tubular acidification dysfunction group in comparison to the normal function group. Female patients in the renal tubular acidification dysfunction group showed significantly decreased femoral neck and total hip BMD and increased susceptibility to fracture. However, there were no such differences in male patients between the two groups.

**Conclusions:**

About 54.6 % of PHPT patients in our study population had abnormal distal renal tubular acidification. PHPT patients with abnormal distal renal tubular acidification may have lower hip bone density. Female PHPT patients with abnormal distal renal tubular acidification showed increased susceptibility to fractures and the development of osteoporosis.

## Background

Chronic acid retention may decrease bone mineral density (BMD). Studies [1, 2] showed that dysfunction of distal renal tubular acidosis may cause bone loss. Patients with abnormal distal renal tubular acidification may not manifest in significant systemic acidosis, however, the increased of acid loading may trigger the release of alkali in the bone, resulting a greater absorption of bone [3]. Sromicki et al. reported that 23 % of osteopoenia/osteoporosis patients may have abnormal distal renal tubular acidification, which may lead to chronic acid retention [4]. Primary hyperparathyroidism (PHPT) is the third most common endocrine disease. Although the incidence of proximal renal tubular acidification dysfunction is known to be higher in PHPT patients, there have been few studies regarding distal renal tubular acidification dysfunction in PHPT [5–7]. Therefore, the aim of the retrospective study was to explore distal renal tubular acidification function in PHPT patients and to examine its potential impact on bone and whether it affects the clinical presentation.

## Methods

### Subjects

The study population consisted of 75 patients, 21 men aged 36–74 years and 54 women aged 29–83 years, with a diagnosis of PHPT [8] according to the following criteria: (1) increased serum calcium above the upper limit of normal range of 2.55 mmol/L, excluding other causes of hypercalcemia; (2) increased PTH above the upper limit of normal range of 7.63 pmol/L; (3) decreased serum phosphorus; and (4) combined with clinical symptom and characteristic radiographic features. The exclusion criteria were as following: patients with multiple endocrine adenoma, secondary or tertiary hyperparathyroidism were excluded from this study. All subjects were hospitalized patients first treated in Tianjin Medical University General Hospital between 2013 and 2019.

Level of Evidence: III,retrospective study.

### Methods

Blood and 24-hour urine electrolytes, kidney and liver function, parathyroid hormone, 25OHD and bone turnover indices were determined. PTH, 25OHD and bone turnover indices were detected by electrochemiluminescence assay in the center laboratory of the hospital. Blood gas analysis and renal tubular acidification were done at the same day. Fasting morning urine was collected and acidification function was determined by the method of titration in an automatic potentiometric Titrator. BMD was measured using a bone densitometer (Lunar Prodigy; GE Healthcare, Waukesha, WI, USA). All patients underwent abdominal and parathyroid ultrasonography. Parathyroid lesions were confirmed by enhanced computed tomography (CT) and radionuclide examination. X-ray examinations of the vertebrae, pelvis, skull and hands were performed in almost all patients. The radiologist reviewed the x-rays to issue the reports. The diagnosis of fragile fractures was based on the x-rays reports and patient’s medical history.

Subjects were divided into the renal tubular acidification dysfunction group and normal function group based on urinary pH>5.8, decreased ammonium ion (normal range 25.84–200 mmol/L) and decreased titratable acid (TA) (normal range 9.57–150 mmol/L) in second morning urine collected after a 12-hour fast. Patients with acute renal dysfunction were excluded from analysis. Acute renal dysfunction [9] is defined as any of the following (Not Graded):increase in SCr by ≥ 0.3 mg/dl (≥ 26.5 umol/l) within 48 hours; or increase in SCr to ≥ 1.5 times baseline, which is known or presumed to have occurred within the prior 7 days; or Urine volume < 0.5 ml/kg/h for 6 hours.

Diagnosis of osteoporosis [10] was based on one or more of the following criteria: (1) fragile fracture of the hip or vertebral body; (2) A value for BMD 2.5 SD or more below the young adult mean (T-score ≤ − 2.5); (3) BMD measurement showing low bone mass (− 2.5< T< −1.0) and fragile fractures of the proximal humerus, pelvis or distal forearm. T-scores are used in postmenopausal women and men aged 50 years or more. For the other populations, Z-scores or fracture risk are considered.

The study was approved by the Institutional Review Board (IRB)/Ethical Committee of Tianjin Medical University General Hospital.

### Statistical methods

Data were analysed using SPSS 25.0 (SPSS, Chicago, IL, USA). The results are presented as the mean ± SD. Analysis of the categorical variables was performed by χ^2^ or Fisher’s test, and comparisons between the two groups were performed using the independent samples *t* test. The continuous variables of non-normal distribution expressed by M (P25, P75) and the non-parametric test were used for comparisons between the groups. In all analyses, *P* <0.05 was taken to indicate statistical significance.

## Results

Gender distribution, age, BMI, course of disease, blood gas analysis indicators, bone turnover indicators, AKP, blood and urine electrolytes (except serum phosphate) were not significantly different between the renal tubular acidification dysfunction group and normal function group. Urine PH was significantly increased, urine ammonium ion and titratable acid were significantly decreased in renal tubular acidification dysfunction group (*P* < 0.01). Serum phosphate was significantly decreased and 25OHD was significantly increased in the renal tubular acidification dysfunction group compared with the normal function group (*P* < 0.05). The renal tubular acidification dysfunction group showed decreased serum potassium level and increased parathyroid hormone (PTH) level but these differences were not significant (both *P* > 0.05). There were no differences in the incidence of kidney stones and fractures between the renal tubular acidification dysfunction group and the normal function group (both *P* > 0.05).(Table 1).

**Table 1:** Comparison of clinical data of the renal tubular acidification dysfunction group and the normal function group (X±S)

Variable	Abnormal acidification group (41)	Normal acidification function group (34)	P
Sex (male/female)	13/28	8/26	0.432 (χ^2^)
Medical history (y)	0.25 (1, 5.5)	0.33 (1, 5)	0.881 (Non-parametric)
Age, y	61.1 ± 12.0	56.8 ± 11.0	0.116
BMI, kg/m^2^	24.2 ± 4.85	24.74 ± 3.84	0.602
Albumin, g/L	41.24 ± 3.86	40.79 ± 3.54	0.604
Albumin-corrected serum calcium, mmol/L	2.80 ± 0.39	2.84 ± 0.31	0.635
Serum phosphate, mmol/L	0.69 ± 0.17	0.79 ± 0.18	0.017
Serum potassium, mmol/L	3.85 ± 0.50	4.08 ± 0.51	0.061
Serum chloride (mmol/L)	107.55 ± 3.45	107.23 ± 3.99	0.717
Serum sodium (mmol/L)	141.06 ± 2.70	142.24 ± 1.71	0.062
Serum magnesium (mmol/L)	0.83 ± 0.11	0.86 ± 0.07	0.220
Serum creatinine, umol/L	58 (47.5, 73)	58 (42.75, 73.25)	0.447 (Non-parametric)
AkP (U/L)	125 (91.5, 231.5)	103.5 (67, 157.75)	0.075 (Non-parametric)
PTH (pmol/L)	59.4 (24.65, 130)	36.1 (17.17, 64.02)	0.061 (Non-parametric)
25OHD (nmol/L)	34.5 (21.4, 64.02)	25.5 (20.45, 32.66)	0.020 (Non-parametric)
Urine PH (4.5-6.5)	6.67±0.45	5.78±0.42	0.000
Urine HCO3^-^(0-12.44mmol/L)	5.50 (3.35, 10.90)	4.50 (2.87, 5.90)	0.231 (Non-parametric)
Urine TA (9.57-150mmol/L)	4.45 ± 2.83	11.91 ± 4.39	0.000
Urine NH4^+^(25.84-200mmol/L)	16.48 ± 5.57	25.62 ± 5.89	0.000
Blood gas PH (7.35-7.45)	7.40 ± 0.28	7.40 ± 0.21	0.266
PCO2 (mmHg)	36.76 ± 5.12	37.51 ± 4.84	0.519
HCO3-(mmol/L)	23.20 ± 3.15	23.87 ± 2.63	0.327
BE (mmol/L)	-1.80 (-3.3, 0.82)	-0.77 (-1.92, 1.00)	0.165 (Non-parametric)
Urinary calcium, (mmol/24h)	8.30 ± 3.30	7.98 ± 3.64	0.280
Urinary phosphate, (mmol/24h)	18.8 ± 7.34	20.4 ± 8.34	0.389
Urinary potassium, (mmol/24h)	18.65 ± 7.11	20.40 ± 8.34	0.331
Urine sodium (mmol/24h)	138.46 (93.01, 205.75)	197.6 (157.59, 228.51)	0.161 (Non-parametric)
Urine magnesium (mmol/24h)	2.85 (1.84, 3.63)	2.7 (2.19, 3.48)	0.898 (Non-parametric)
OC (ng/ml)	60.06 (29.49, 125.25)	59.61 (33.58, 131.30)	0.903 (Non-parametric)
CTX (ng/ml)	0.99 (0.65, 1.86)	1.18 (0.80, 2.49)	0.225 (Non-parametric)
PINP (ng/ml)	70 (45.28, 125.25)	74.28 (41.09, 103.80)	0.709 (Non-parametric)
Kidney stones (with/without)	19/22	12/22	0.333 (χ^2^)
Fracture (with/without)	13/28	10/24	0.830 (χ^2^)

Total hip BMD was significantly decreased in the renal tubular acidification dysfunction group compared with the normal function group (*P* < 0.05). There were no differences in the proportions of patients with osteoporosis at any site between the two groups (*P* > 0.05). (Table 2).

**Table 2:** Comparison of bone mineral density in the renal tubular acidification dysfunction group and the normal function group (X±S)

Variable (BMD)	Distal renal tubular dysfunction group	Distal renal tubular normal group	P
L1-L4(g/cm^2^)	0.837 ± 0.206	0.923 ± 0.210	0.166
Femoral neck (g/cm^2^)	0.661 ± 0.173	0.727 ± 0.130	0.112
Total hip (g/cm^2^)	0.676 ± 0.177	0.770 ± 0.139	0.030
Osteoporosis at any site (n, %)	23(62.1%)	11(44%)	0.159 (χ^2^)

On stratification according to sex, serum phosphate level, femoral neck and total hip BMD were significantly decreased, while the proportion of patients with bone fractures and osteoporosis at any site were significantly increased in female patients in the renal tubular acidification dysfunction group compared with the normal function group. However, there were no such differences in male patients between the two groups. The comparison of the other parameters between the two groups was the same as before the gender stratification. (Tables 3 and 4)

**Table 3:** Comparison of bone mineral density between female patients in the renal tubular acidification dysfunction group and the normal function group (X±S)

	Distal renal tubular dysfunction group (28)	Distal renal tubular normal group (26)	P
Age, y	60.21 ± 13.4	57.6 ± 11.2	0.436
BMI (kg/m^2^)	23.82 ± 5.16	24.19 ± 3.93	0.770
Premenopausal/postmenopausal	4/24	3/23	0.112
Albumin, g/L	40.89 ± 4.11	40.50 ± 3.56	0.710
Albumin-corrected serum calcium, mmol/L	2.80 ± 0.42	2.85 ± 0.31	0.615
Serum phosphate, mmol/L	0.71 ± 0.19	0.82 ± 0.18	0.034
Serum sodium (mmol/L)	140.92 ± 3.05	141.7 ± 2.63	0.371
Serum magnesium (mmol/L)	0.81 ± 0.12	0.86 ± 0.08	0.149
PTH (pmol/L)	64.9 (25.1, 145.25)	30.15 (18.95, 65.92)	0.083 (Non-parametric)
25OHD (nmol/L)	29.5 (20.4, 41.8)	24.99 (20.23, 32.61)	0.272 (Non-parametric)
Urinary calcium, mmol/24h	8.01 ± 3.13	9.86 ± 6.21	0.169
Urinary phosphate, mmol/24h	17.84 ± 6.15	19.51 ± 8.85	0.421
Urine sodium (mmol/24h)	159.12 (97.65, 208)	187.2 (101.4, 223.0)	0.683 (Non-parametric)
Urine magnesium (mmol/24h)	3.3 (2.17, 4.06)	2.7 (2.19, 3.50)	0.486 (Non-parametric)
L1-L4 (g/cm^2^)	0.787 ± 0.195	0.863 ± 0.187	0.214
Femoral neck (g/cm^2^)	0.604 ± 0.163	0.719 ± 0.129	0.023
Total hip (g/cm^2^)	0.604 ± 0.159	0.756 ± 0.128	0.002
Fracture (with/without)	12/16	4/22	0.038 (Fisher)
Kidney stones (with/without)	11/17	5/21	0.107 (χ^2^)
Osteoporosis at any site (n,%)	19 (67%)	9 (34%)	0.015 (χ^2^)

**Table 4:** Comparison of bone mineral density between male patients in the renal tubular acidification dysfunction group and the normal function group (X±S)

	Distal renal tubular dysfunction group (13)	Distal renal tubular normal group (8)	P
Age, y	63.5 ± 8.7	54.25 ± 13.7	0.081
BMI (kg/m^2^)	24.94 ± 4.2	26.51 ± 3.09	0.373
Albumin, g/L	42.25 ± 3.30	41.75 ± 3.49	0.750
Albumin-corrected serum calcium, mmol/L	2.80 ± 0.32	2.79 ± 0.34	0.968
Serum phosphate, mmol/L	0.66 ± 0.13	0.72 ± 0.17	0.402
PTH (pmol/L)	54.15 (25.2, 112.75)	41.25 (15.52, 62.8)	0.217 (Non-parametric)
25OHD (nmol/L)	40.05 (20.0, 48.1)	31.4 (23.8, 32.4)	0.554 (Non-parametric)
Serum sodium (mmol/L)	141.9 ± 1.28	141.85 ± 1.95	0.957
Serum magnesium (mmol/L)	0.87 ± 0.07	0.86 ± 0.07	0.866
Urinary calcium, mmol/24h	9.07 ± 3.83	9.23 ± 6.37	0.944
Urinary phosphate, mmol/24h	20.56 ± 9.25	23.28 ± 5.99	0.473
Urine sodium (mmol/24h)	110.61 (68.41, 183.39)	209.8 (141.18, 259.26)	0.093 (Non-parametric)
Urine magnesium (mmol/24h)	2.12 (1.52, 3.49)	2.82 (2.23, 4.39)	0.258 (Non-parametric)
L1-L4 (g/cm^2^)	1.004 ± 0.165	1.076 ± 0.213	0.441
Femoral neck (g/cm^2^)	0.797 ± 0.118	0.781 ± 0.133	0.798
Total hip (g/cm^2^)	0.823 ± 0.134	0.835 ± 0.163	0.870
Kidney stones (with/without)	3/10	2/6	1.000 (Fisher)
Fracture (with/without)	8/5	7/1	0.336 (Fisher)
Osteoporosis at any site(n,%)	3(23%)	1(12.5%)	0.549 (Fisher)

## Discussion

Abnormal renal tubular acidosis may affect bone metabolism. Results of former study [3] showed that patients with abnormal distal renal tubular acidification may not manifest in significant systemic acidosis, this persistent acid load may trigger the release of the alkali in the bone, resulting in greater bone loss. Other study found that patients with osteoporosis or reduced BMD have a strong relationship with incomplete distal renal tubular acidosis [11] .It has been reported that patients with PHPT are prone to renal tubular dysfunction [12, 13]. So we carried out this retrospective study try to find whether the dysfunction of renal tubular acidification may affect the PHPT patients’ outcomes. The cut-off rate we used to define acidification function is from our lab. We found about half of the PHPT patients (54.6 %) had distal renal tubular acidification dysfunction. Although the impaired titratable acid and ammonia ion secretion in the distal renal tubules did not cause body acid-base imbalance, we found that total hip bone density was significantly decreased in the patients with this abnormal acidification. Stratification according to gender showed that female patients with abnormal distal tubular acidification function had lower femur neck and total hip BMD and were more susceptible to bone fracture compared to those in the normal function group. However, there were no such differences in male patients between the two groups. Castellano et al. [14, 15] reported that sex and menopausal status may affect the clinical presentations of PHPT. They found that postmenopausal female PHPT patients were more prone to osteoporosis compared with male patients. Cheng et al. [16] reported that sex and age can modify skeletal manifestations in an experimental model of hyperparathyroidism, and found that high PTH levels reduced trabecular bone mass in female mice but had the opposite effect in male mice. Our results were consistent with these studies, in the condition of increasing acid loading, female PHPT patients appeared to have increased susceptibility to osteoporosis and bone fracture.

With the exception of serum phosphate and plasma 25OHD levels, serum calcium, alkaline phosphatase, PTH, bone turnover indices and electrolyte in urine were similar between the groups with and without acidification disorders. Therefore, we speculated that decreased secretion of acid in the distal renal tubular may directly affect bone mass.

The pathogenesis of distal renal tubular acidification dysfunction in PHPT patients is not clear. It has been suggested that hypercalciuric renal tubular damage may induce dysfunction of the distal renal tubular epithelium [6, 12]. However, we found no significant differences in urinary calcium levels between the groups with and without acidification dysfunction in the present study. Other groups have suggested that the long duration of the disease is responsible for renal interstitial calcification, which causes tubular acidification dysfunction [6, 11]. However, we found no correlation between acidification dysfunction and the duration of the disease. Further studies are required to determine the mechanism underlying distal renal tubular acidification dysfunction in PHPT.

## Conclusion

In summary, our study found PHPT patients with abnormal distal renal tubular acidification may have lower hip bone density. Female PHPT patients with distal renal tubular acidification dysfunction showed increased susceptibility to fractures and the development of osteoporosis.

Our study is a real-world study, it is retrospectively analysis the data from the hospitalized patients. The test to evaluate the renal tubular acidification has been used in the Nephrology Department of our hospital for more than 20 years. However this method is not standardized and wildly accepted. The relatively small- scale of this study may affect its strength. So further larger scale and multi-center study are needed to confirm our findings.

## Data Availability

The datasets used and/or analysed during the current study available from the corresponding author on reasonable request.

## Abbreviations

25OHD: 25 hydroxyvitamin D
PHPT: Primary hyperparathyroidism
PTH: Parathyroid hormone
BMD: Bone mineral density
AKP: Alkaline phosphatase
TA: Titratable acid
OC: Osteocalcin
CTX: Type I collagen cross-linked C-telopeptide
PINP: Procollagen type l N-terminal propeptide
